# The psychometric properties of motors of COVID-19 vaccination acceptance scale (MoVac-COVID19S): A dataset across five regions

**DOI:** 10.1016/j.dib.2022.108103

**Published:** 2022-03-27

**Authors:** Daniel Kwasi Ahorsu, Chung-Ying Lin, I-Hua Chen, Irfan Ullah, Sheikh Shoib, Shafi Ullah Zahid, Emma Sethina Adjaottor, Frimpong-Manso Addo, Amir H Pakpour

**Affiliations:** a Department of Rehabilitation Sciences, The Hong Kong Polytechnic University, Hung Hom, Hong Kong; b Institute of Allied Health Sciences, College of Medicine, National Cheng Kung University, Tainan, Taiwan; c Chinese Academy of Education Big Data, Qufu Normal University, Qufu City, Shandong, China; d Kabir Medical College, Gandhara University, Peshawar, Pakistan; e Sheikh Shoib Department of Psychiatry, Jawahar Lal Nehru Memorial Hospital, Srinagar, India; f Department of Neurosurgery, Jamhuriat hospital, Kabul, Afghanistan; g Department of Behavioural Sciences, Kwame Nkrumah University of Science and Technology, Ghana; h Department of Nursing, School of Health and Welfare, Jönköping University, Jönköping, Sweden

**Keywords:** COVID-19, Taiwan, China, Ghana, India, Afghanistan, Measurement Invariance

## Abstract

The novel coronavirus disease 2019 (COVID-19) continues to plague the world. Hence, there is been an effort to mitigate this virus and its effects with several means including vaccination which is one of the most effective ways of controlling the virus. However, efforts at getting people to vaccinate have met several challenges. To help with understanding the reasons underlying an individual's willingness to take COVID-19 vaccine or not, a scale called Motors of COVID-19 Vaccination Acceptance Scale (MoVac-COVID19S) was developed. To expand its usability worldwide (as it has currently been limited to only China and Taiwan), data were collected in other countries (regions) too. Therefore, this MoVac-COVID19S data is from five countries (that is, India, Ghana, Afghanistan, Taiwan, and mainland China) which cut across five regions. A total of 6053 participants across the stated countries completed the survey between January and March 2021 using a cross-sectional survey design. The different sections of the survey solicited sociodemographic information (e.g., country, age, gender, educational level, and profession) and the MoVac-COVID19S data from the participants. The data collected from this survey were analyzed using descriptive statistics, which were carried out using the IBM SPSS version 22.0.

## Specifications Table

**Table:** 

Subject	Health and medical sciences
Specific subject area	Public Health and Health Policy
Type of data	Table
How the data were acquired	Data were collected using survey (paper-and-pencil or online) method where participants completed the form. A copy of the survey is included as a Supplementary File.
Data format	Raw, Analyzed
Description of data collection	A total of 6053 adult participants across five countries (India, Ghana, Afghanistan, Taiwan, and mainland China) completed the survey between January and March 2021 using a cross-sectional survey design. The sections of the survey solicited sociodemographic information (e.g., country, age, gender, educational level, and profession) and the MoVac-COVID19S (also named as DrVac-COVID19S) data from the participants.
Data source location	• Jawahar Lal Nehru Memorial Hospital, Srinagar, India • Kwame Nkrumah University of Science and Technology, Ghana • Jamhuriat hospital, Kabul, Afghanistan • National Cheng Kung University, Tainan, Taiwan • Qufu Normal University, Qufu City, Shandong, China
Data accessibility	Repository name: Harvard Dataverse Data identification number: https://doi.org/10.7910/DVN/U8ZYDF Direct URL to data: https://dataverse.harvard.edu/dataset.xhtml?persistentId=doi:10.7910/DVN/U8ZYDF Questionnaire: https://dataverse.harvard.edu/dataset.xhtml?persistentId=doi:10.7910/DVN/U8ZYDF
Related research article	Y.-C. Yeh, I.H. Chen, D.K. Ahorsu, N.-Y. Ko, K.-L. Chen, P.-C. Li, C.-F. Yen, C.-Y. Lin, M.D. Griffiths, A.H. Pakpour, Measurement Invariance of the Drivers of COVID-19 Vaccination Acceptance Scale: Comparison between Taiwanese and Mainland Chinese-Speaking Populations, Vaccines 9(3) (2021) 297. https://doi.org/10.3390/vaccines9030297

## Value of the Data

• This data is useful as it comprises data from a largescale survey across five regions/countries worldwide on COVID-19 vaccination acceptance. Hence, the data can always be used to verify the psychometric properties of the MoVac-COVID19S (COVID-19 vaccination acceptance scale; also named as DrVac-COVID19S) and its suitability for use worldwide.
• The data can be beneficial to the following group of persons: Researchers who are interested in communicable disease, psychometrics, health promotion, health psychology, public health, epidemiology, and health behavior as the findings from this dataset will serve as the basis for assessing citizens’ willingness to take COVID-19 vaccination.
• The data may be useful for researchers who want to replicate or extend the psychometric properties (especially, measurement invariance) of the MoVac-COVID19S by adding their country's data to this data.

## 1 Data Description

The challenges posed by COVID-19 continues unabated [1], [2], [3], [4], [5], [6], [7], [8], [9]. One of the most efficient ways of controlling this pandemic is by vaccination. However, the vaccination drive has met lots of challenges. The Motors of COVID-19 Vaccination Acceptance Scale (MoVac-COVID19S; also named as Drivers of COVID-19 Vaccination Acceptance Scale [DrVac-COVID19S]) was, therefore, developed to help with understanding the reasons underlying an individual's willingness to take COVID-19 vaccine or not. To expand its usability worldwide (as it has currently been limited to only China and Taiwan), data were collected in other countries (regions) too. Therefore, the MoVac-COVID19S data is from five countries (that is, India, Ghana, Afghanistan, Taiwan, and mainland China) which cut across five regions. A total of 6053 participants across the above-stated countries completed the survey between January and March 2021 using a cross-sectional survey design. The sections of the survey solicited sociodemographic information (e.g., country, age, gender, educational level, and profession) and the MoVac-COVID19S data from the participants (please find the questionnaire in the supplementary material). Table 1 shows the sociodemographic characteristics of the participants while Table 2 shows the distributions of responses related to the items of the MoVac-COVID19S. For the demographic characteristics, the codes used were 0 and 1 for gender (females and males respectively), 1, 2, and 3 for educational levels (others, undergraduate, and postgraduate respectively), and 0 and 1 for profession (not health related and health related respectively). For the MoVac-COVID19S, the codes 1, 2, 3, 4, 5, 6, 7 were used to represent Strongly Disagree, Disagree, Slightly Disagree, Neither Disagree nor Agree, Slightly Agree, Agree, and Strongly Agree respectively. For group (countries), 1, 2, 3, 4, and 5 were used as codes for Taiwan, mainland China, India, Ghana, and Afghanistan respectively.

**Table 1:** Distribution of responses in relation to sociodemographic variables.

Socio-demographics	Taiwan (n=932; 15.4%)	China (n=3145; 52%)	India (n=508; 8.4%)	Ghana (n=1244; 20.6%)	Afghanistan (n=224; 3.7%)	Total (n=6053; 100%)
Age; Mean±SD	25.39±6.46	20.84±2.67	24.46±7.34	20.34±1.75	26.82±4.76	22.00±4.56
<30 years	805 (14.1%)	2935 (51.3%)	385 (6.7%)	1132 (19.8%)	168 (2.9%)	5425 (94.7%)
≥30 years	125 (2.2%)	45 (0.8%)	83 (1.4%)	1 (0%)	47 (0.8%)	301 (5.3%)

Gender						
Male	354 (5.9%)	1567 (26.2%)	176 (2.9%)	789 (13.2%)	144 (2.4%)	3030 (50.6%)
Female	578 (9.7%)	1578 (26.4%)	328 (5.5%)	393 (6.6%)	80 (1.3%)	2957 (49.4%)

Educational Level						
Others	0 (0%)	31 (0.5%)	67 (1.2%)	147 (2.6%)	NA	245 (4.3%)
Undergraduate	595 (10.3%)	3026 (52.6%)	215 (3.7%)	988 (17.2%)	NA	4824 (83.9%)
Postgraduate	337 (5.9%)	88 (1.5%)	226 (3.9%)	32 (0.6%)	NA	683 (11.9%)

Profession						
Not Health related	468 (7.9%)	2904 (49.1%)	432 (7.3%)	1043 (17.6%)	0 (0%)	4847 (82.0%)
Health related	403 (6.8%)	241 (4.1%)	76 (1.3%)	121 (2.%)	224 (3.8%)	1065 (18.0%)

**Table 2:** Distribution of responses related to MoVac-COVID19S.

Items		Frequency	Percentages
1. Vaccination is a very effective way to protect me against the COVID-19.	Strongly Disagree	143	2.4
	Disagree	88	1.5
	Slightly disagree	249	4.1
	Neither disagree nor agree	826	13.6
	Slightly agree	1291	21.3
	Agree	1753	29.0
	Strongly Agree	1703	28.1

2. I know very well how vaccination protects me from the COVID-19.	Strongly Disagree	182	3.0
	Disagree	132	2.2
	Slightly disagree	303	5.0
	Neither disagree nor agree	890	14.7
	Slightly agree	1313	21.7
	Agree	1538	25.4
	Strongly Agree	1695	28.0

3. It is important that I get the COVID-19 jab.	Strongly Disagree	190	3.1
	Disagree	110	1.8
	Slightly disagree	210	3.5
	Neither disagree nor agree	899	14.9
	Slightly agree	1018	16.8
	Agree	1613	26.6
	Strongly Agree	2013	33.3

4. Vaccination greatly reduces my risk of catching COVID-19.	Strongly Disagree	186	3.1
	Disagree	93	1.5
	Slightly disagree	216	3.6
	Neither disagree nor agree	769	12.7
	Slightly agree	1117	18.5
	Agree	1742	28.8
	Strongly Agree	1930	31.9

5. I understand how the COVID-19 jab helps my body fight the COVID-19 virus.	Strongly Disagree	190	3.1
	Disagree	154	2.5
	Slightly disagree	303	5.0
	Neither disagree nor agree	940	15.5
	Slightly agree	1273	21.0
	Agree	1557	25.7
	Strongly Agree	1636	27.0

6. The COVID-19 jab plays an important role in protecting my life and that of others.	Strongly Disagree	157	2.6
	Disagree	98	1.6
	Slightly disagree	188	3.1
	Neither disagree nor agree	773	12.8
	Slightly agree	1016	16.8
	Agree	1820	30.1
	Strongly Agree	2001	33.1

7. I feel under pressure to get the COVID-19 jab.	Strongly Disagree	526	8.7
	Disagree	333	5.5
	Slightly disagree	506	8.4
	Neither disagree nor agree	1331	22.0
	Slightly agree	1322	21.8
	Agree	1048	17.3
	Strongly Agree	987	16.3

8. The contribution of the COVID-19 jab to my health and well-being is very important.	Strongly Disagree	165	2.7
	Disagree	88	1.5
	Slightly disagree	175	2.9
	Neither disagree nor agree	898	14.8
	Slightly agree	1110	18.3
	Agree	1761	29.1
	Strongly Agree	1856	30.7
9. I can choose whether to get a COVID-19 jab or not.	Strongly Disagree	201	3.3
	Disagree	101	1.7
	Slightly disagree	158	2.6
	Neither disagree nor agree	891	14.7
	Slightly agree	973	16.1
	Agree	1662	27.5
	Strongly Agree	2067	34.1

10. How the COVID-19 jab works to protect my health is a mystery to me.	Strongly Disagree	503	8.3
	Disagree	414	6.8
	Slightly disagree	482	8.0
	Neither disagree nor agree	1204	19.9
	Slightly agree	1306	21.6
	Agree	1041	17.2
	Strongly Agree	1103	18.2

11. I get the COVID-19 jab only because I am required to do so.	Strongly Disagree	590	9.7
	Disagree	481	7.9
	Slightly disagree	657	10.9
	Neither disagree nor agree	1389	22.9
	Slightly agree	1117	18.5
	Agree	847	14.0
	Strongly Agree	972	16.1

12. Getting the COVID-19 jab has a positive influence on my health.	Strongly Disagree	222	3.7
	Disagree	130	2.1
	Slightly disagree	254	4.2
	Neither disagree nor agree	1336	22.1
	Slightly agree	1289	21.3
	Agree	1415	23.4
	Strongly Agree	1407	23.2

## 2 Experimental Design, Materials and Methods

The data was collected using a survey with a cross-sectional design. The Taiwanese participants were recruited using snowballing method via the posts on social media pages and social networking apps. A total of 932 questionnaires were collected between January 5 and February 5, 2021. The MoVac-COVID19S for the Taiwanese was written in traditional Chinese, Taiwan's official language. Similarly, snowballing method (posts on social media pages and social networking apps (e.g., WeChat)) were used to recruit Mainland Chinese participants. A total of 3145 questionnaires were collected between January 5 and January 16, 2021. The MoVac-COVID19S for mainland Chinese was written in simplified Chinese, mainland China's official language. Also, snowballing method (posts on social media pages and social networking apps (e.g., Facebook and Whatsapp)) were used to recruit Indian participants. A total of 508 questionnaires were collected between July 25 and October 5, 2021. The MoVac-COVID19S for the Indians was written in English, the official language of Indian universities. The snowballing method (sharing the survey link in various Afghanistan healthcare workers’ groups (e.g., Fan page in the Facebook)) was also used to recruit Afghan participants. A total of 224 questionnaires were collected between April 1 and July 31, 2021. The MoVac-COVID19S used for the Afghans was written in English. Among the Ghanaian participants, a convenient sampling strategy was used to recruit the participants. A total of 1,244 questionnaires between January 25 and March 12, 2021. The MoVac-COVID19S for Ghanaians was written in English, the official language of Ghanaians. All the recruited participants are natives of their respective countries [10]. All the participants consented to participate by providing a written Informed consent (signing or ticking). The data were analyzed using descriptive statistics (specifically, Mean and Standard Deviation, and frequency with percentage) using the IBM SPSS 22.0.

The MoVac-COVID19S was developed from an original MoVac-Flu Scale by changing the word from “flu” to “COVID-19” to derive the current form of MoVac-COVID19S with the kind permission of MoVac-Flu Scale's developer, Professor Vallée-Tourangeau [11,12]. Hence, the cognitive model of empowerment (CME) that formed the basis of the MoVac-Flu Scale remains unchanged under the MoVac-COVID19S [13]. The MoVac-COVID19S, therefore, is made up of 12 items; nine positively worded items and three negatively worded items. It must be noted that previous evidence indicated that the MoVac-COVID19S has wording effects that should be taken into account when testing its factor structure [11,14]. The items of MoVac-COVID19S are rated on a 7-point Likert scale response format (from Strongly disagree =1 to Strongly agree =7). The participant's responses are summed together to get a total score after aligning the directions of the positively and negatively worded items. Hence, the higher the total score (including the entire instrument and the four domains), the higher the levels of acceptance to get COVID-19 vaccinated. Both the 9-item version (ω = 0.921) and the 12-item version (ω = 0.898) have high internal consistency.

## Ethics Statements

The data was collected in conformity with the Helsinki declaration (1975) and with ethical approvals from each of the respective countries. The ethical approval was granted by the Kaohsiung Medical University Chung-Ho Memorial Hospital (IRB ref: KMUHIRB-EXEMPT(I)-20200019) in Taiwan, the Jianxi Psychological Consultant Association (IRB ref: JXSXL-2020-DE22) in mainland China, the University of Kashmir's Department of Social Work (IRB ref: F-2(MSW) KU/2021dated 22-6-2021) in India, The Kwame Nkrumah University of Science and Technology (IRB ref: CHRPE/AP/283/21) in Ghana, and the Kateb University Hospital Ethics Committee (IRB ref: 2305) in Afghanistan. Also, informed consents were obtained from all the participants before data collection.

## CRediT Author Statement

**Daniel Kwasi Ahorsu:** Writing - Original draft preparation, Software; **Chung-Ying Lin:** Supervision; **Amir H Pakpour:** Conceptualization, Methodology, Software; **Emma Sethina Adjaottor, Frimpong-Manso Addo, I-Hua Chen, Irfan Ullah:** Visualization, Investigation; **Sheikh Shoib:** Visualization, Investigation; **Shafi Ullah Zahid:** Visualization, Investigation.

## Declaration of Competing Interest

The authors declare that they have no known competing financial interests or personal relationships that could have appeared to influence the work reported in this paper.

## Data Availability

The psychometric properties of Motors of COVID-19 Vaccination Acceptance Scale (MoVac-COVID19S) (Original data).
